# {4,4′-Dibromo-6,6′-dimeth­oxy-2,2′-[1,2-phenyl­enebis(nitrilo­methanylyl­idene)]-κ^4^ *O* ^1^,*N*,*N*′,*O* ^1′^}nickel(II)

**DOI:** 10.1107/S1600536812004321

**Published:** 2012-02-10

**Authors:** Yongling Sun

**Affiliations:** a Department of Biology, Dezhou University, Dezhou 253023, People’s Republic of China

## Abstract

In the title complex, [Ni(C_22_H_16_Br_2_N_2_O_4_)], the Ni^II^ ion is coordinated by two N atoms and two O atoms of a tetra­dentate Schiff base ligand, forming a slightly distorted square-planar coordination environment. The dihedral angle between the two bromo-substituted benzene rings is 10.1 (3)°.

## Related literature

For Schiff base ligands in coordination chemistry, see: Ghosh *et al.* (2006 ▶); Nayak *et al.* (2006 ▶). For related structures, see: Wang *et al.* (1994 ▶); Bhattacharya *et al.* (2011 ▶); Yu *et al.* (2009 ▶); Kargar *et al.* (2009 ▶); Felices *et al.* (2009 ▶).
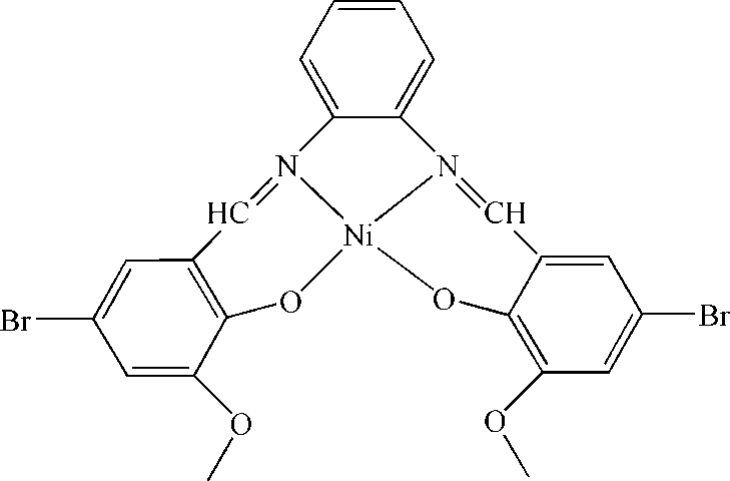


## Experimental

### 

#### Crystal data

[Ni(C_22_H_16_Br_2_N_2_O_4_)]
*M* *_r_* = 590.90
Monoclinic, 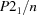

*a* = 15.288 (7) Å
*b* = 8.213 (3) Å
*c* = 16.473 (7) Å
β = 90.171 (8)°
*V* = 2068.3 (15) Å^3^
*Z* = 4
Mo *K*α radiation
μ = 4.84 mm^−1^
*T* = 293 K
0.17 × 0.15 × 0.12 mm

#### Data collection

Bruker APEXII diffractometer
Absorption correction: multi-scan (*SADABS*; Sheldrick, 1996 ▶) *T*_min_ = 0.494, *T*_max_ = 0.595
9492 measured reflections
3613 independent reflections
2628 reflections with *I* > 2σ(*I*)
*R*_int_ = 0.088

#### Refinement

*R*[*F* ^2^ > 2σ(*F* ^2^)] = 0.065
*wR*(*F* ^2^) = 0.170
*S* = 1.02
3613 reflections
282 parameters
H-atom parameters constrained
Δρ_max_ = 1.61 e Å^−3^
Δρ_min_ = −1.16 e Å^−3^

### 

Data collection: *APEX2* (Bruker, 2004 ▶); cell refinement: *SAINT* (Bruker, 2001 ▶); data reduction: *SAINT*; program(s) used to solve structure: *SHELXS97* (Sheldrick, 2008 ▶); program(s) used to refine structure: *SHELXL97* (Sheldrick, 2008 ▶); molecular graphics: *SHELXTL* (Sheldrick, 2008 ▶); software used to prepare material for publication: *SHELXTL*.

## Supplementary Material

Crystal structure: contains datablock(s) I, global. DOI: 10.1107/S1600536812004321/lh5411sup1.cif

Structure factors: contains datablock(s) I. DOI: 10.1107/S1600536812004321/lh5411Isup2.hkl

Additional supplementary materials:  crystallographic information; 3D view; checkCIF report

## Figures and Tables

**Fig. 1.**
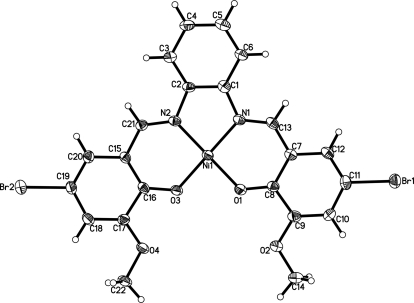
The molecular structure. Displacement ellipsoids are drawn at the 30% probability level.

## supplementary crystallographic information

### Comment

Schiff-bases ligands, especially for those which are chelating, play an important role in the development of coordination chemistry as they readily form stable complexes with most transition metals (Ghosh *et al.*, 2006; Nayak *et al.*, 2006). Here, we present the structure of a new Ni(II) complex based on the tetradentate chelating Schiff-base ligand 1,2-diaminobenzene-*N*,*N*'-bis (5-bromo-3-methoxysalicylideneimine).

The molecular structure of the title complex is shown in Figure 1. The coordination of the Ni^II^ ion is slightly distorted square-planar, formed by two N atoms and two O atoms of the Schiff-base ligand. The mean deviation from the plane formed by the two N atoms, two O atoms and the Ni^II^ ion is 0.0426 Å. The Ni—N and Ni—O bond lengths are consistent with the corresponding distances in other nickel(II) complexes containing similar chelating tetradentate schiff-base ligands (Wang *et al.*, 1994; Bhattacharya *et al.*, 2011; Yu *et al.*, 2009; Kargar *et al.*, 2009; Felices *et al.*, 2009).

### Experimental

The Schiff-base ligand can be readily synthesized by condensation 1,2-diaminobenzene and 5-bromo-2-hydroxy-3-methoxybenzaldehyde with the ratio 1:2 in ethanol. The preparation of the title complex was carried out by the reaction of Ni(ClO_4_)_2_.6H_2_O and the schiff-base ligand (1:1, molar ratio) in methanol. After the stirring process was continued for about half an hour at room temperature, the mixture was filtered and the filtrate was allowed to slowly evaporate in air for several days to produce crystals suitable for X-ray diffraction with a yield about 56%.

### Refinement

H atoms were placed in calculated positions with C—H distances of 0.93 and 0.96 Å, and were allowed for as riding atoms with *U*_iso_(H) = 1.2*U*_eq_(C) or 1.5*U*_eq_(C_methyl_).

### Crystal data

**Table:**

[Ni(C_22_H_16_Br_2_N_2_O_4_)]	*F*(000) = 1168
*M**_r_* = 590.90	*D*_x_ = 1.898 Mg m^−^^3^
Monoclinic, *P*2_1_/*n*	Mo *K*α radiation, λ = 0.71073 Å
Hall symbol: -P 2yn	Cell parameters from 2246 reflections
*a* = 15.288 (7) Å	θ = 2.5–25.7°
*b* = 8.213 (3) Å	µ = 4.84 mm^−^^1^
*c* = 16.473 (7) Å	*T* = 293 K
β = 90.171 (8)°	Block, red-brown
*V* = 2068.3 (15) Å^3^	0.17 × 0.15 × 0.12 mm
*Z* = 4	

### Data collection

**Table:**

Bruker APEXII diffractometer	3613 independent reflections
Radiation source: fine-focus sealed tube	2628 reflections with *I* > 2σ(*I*)
Graphite monochromator	*R*_int_ = 0.088
φ and ω scans	θ_max_ = 25.0°, θ_min_ = 1.8°
Absorption correction: multi-scan (*SADABS*; Sheldrick, 1996)	*h* = −15→18
*T*_min_ = 0.494, *T*_max_ = 0.595	*k* = −9→9
9492 measured reflections	*l* = −19→17

### Refinement

**Table:**

Refinement on *F*^2^	Primary atom site location: structure-invariant direct methods
Least-squares matrix: full	Secondary atom site location: difference Fourier map
*R*[*F*^2^ > 2σ(*F*^2^)] = 0.065	Hydrogen site location: inferred from neighbouring sites
*wR*(*F*^2^) = 0.170	H-atom parameters constrained
*S* = 1.02	*w* = 1/[σ^2^(*F*_o_^2^) + (0.1048*P*)^2^] where *P* = (*F*_o_^2^ + 2*F*_c_^2^)/3
3613 reflections	(Δ/σ)_max_ = 0.001
282 parameters	Δρ_max_ = 1.61 e Å^−^^3^
0 restraints	Δρ_min_ = −1.16 e Å^−^^3^

### Special details

**Table:**

Geometry. All e.s.d.'s (except the e.s.d. in the dihedral angle between two l.s. planes) are estimated using the full covariance matrix. The cell e.s.d.'s are taken into account individually in the estimation of e.s.d.'s in distances, angles and torsion angles; correlations between e.s.d.'s in cell parameters are only used when they are defined by crystal symmetry. An approximate (isotropic) treatment of cell e.s.d.'s is used for estimating e.s.d.'s involving l.s. planes.
Refinement. Refinement of *F*^2^ against ALL reflections. The weighted *R*-factor *wR* and goodness of fit *S* are based on *F*^2^, conventional *R*-factors *R* are based on *F*, with *F* set to zero for negative *F*^2^. The threshold expression of *F*^2^ > σ(*F*^2^) is used only for calculating *R*-factors(gt) *etc*. and is not relevant to the choice of reflections for refinement. *R*-factors based on *F*^2^ are statistically about twice as large as those based on *F*, and *R*- factors based on ALL data will be even larger.

### Fractional atomic coordinates and isotropic or equivalent isotropic displacement parameters (Å^2^)

**Table:**

	*x*	*y*	*z*	*U*_iso_*/*U*_eq_	
Ni1	0.03371 (5)	0.81849 (9)	0.06315 (4)	0.0308 (3)	
Br1	0.20569 (5)	0.28032 (9)	−0.25514 (4)	0.0469 (3)	
Br2	0.01289 (5)	1.43548 (10)	0.38330 (5)	0.0626 (3)	
O1	0.1366 (3)	0.7166 (5)	0.0303 (3)	0.0393 (11)	
O2	0.2972 (3)	0.6233 (6)	0.0006 (3)	0.0521 (13)	
O3	0.1011 (3)	0.9012 (5)	0.1472 (2)	0.0376 (10)	
O4	0.2250 (3)	1.0180 (5)	0.2414 (3)	0.0446 (12)	
N1	−0.0338 (3)	0.7335 (6)	−0.0210 (3)	0.0325 (12)	
N2	−0.0688 (3)	0.9208 (6)	0.0960 (3)	0.0326 (12)	
C1	−0.1227 (4)	0.7828 (7)	−0.0186 (4)	0.0368 (15)	
C2	−0.1425 (4)	0.8846 (7)	0.0455 (4)	0.0337 (14)	
C3	−0.2253 (4)	0.9459 (8)	0.0556 (4)	0.0434 (17)	
H3	−0.2377	1.0151	0.0988	0.052*	
C4	−0.2910 (5)	0.9029 (10)	0.0002 (4)	0.0535 (19)	
H4	−0.3474	0.9436	0.0061	0.064*	
C5	−0.2714 (5)	0.7999 (9)	−0.0633 (4)	0.0500 (18)	
H5	−0.3151	0.7710	−0.0999	0.060*	
C6	−0.1887 (4)	0.7393 (8)	−0.0735 (4)	0.0404 (15)	
H6	−0.1765	0.6698	−0.1165	0.048*	
C7	0.0816 (4)	0.5844 (7)	−0.0892 (3)	0.0327 (14)	
C8	0.1486 (4)	0.6307 (7)	−0.0341 (4)	0.0334 (14)	
C9	0.2345 (4)	0.5739 (8)	−0.0532 (4)	0.0394 (16)	
C10	0.2505 (4)	0.4733 (8)	−0.1171 (4)	0.0373 (15)	
H10	0.3069	0.4355	−0.1268	0.045*	
C11	0.1814 (5)	0.4272 (7)	−0.1684 (4)	0.0388 (16)	
C12	0.0991 (4)	0.4797 (7)	−0.1560 (4)	0.0368 (15)	
H12	0.0541	0.4480	−0.1907	0.044*	
C13	−0.0056 (4)	0.6388 (7)	−0.0782 (4)	0.0358 (15)	
H13	−0.0468	0.6029	−0.1158	0.043*	
C14	0.3853 (5)	0.5783 (12)	−0.0163 (6)	0.079 (3)	
H14A	0.3907	0.4619	−0.0148	0.118*	
H14B	0.4235	0.6256	0.0236	0.118*	
H14C	0.4012	0.6171	−0.0692	0.118*	
C15	−0.0078 (4)	1.0701 (7)	0.2093 (4)	0.0317 (14)	
C16	0.0768 (4)	1.0105 (7)	0.1998 (4)	0.0332 (14)	
C17	0.1444 (4)	1.0825 (7)	0.2515 (4)	0.0363 (15)	
C18	0.1245 (5)	1.2049 (8)	0.3053 (4)	0.0429 (17)	
H18	0.1681	1.2509	0.3374	0.051*	
C19	0.0388 (5)	1.2591 (8)	0.3112 (4)	0.0413 (16)	
C20	−0.0283 (4)	1.1942 (7)	0.2665 (4)	0.0371 (15)	
H20	−0.0855	1.2302	0.2732	0.045*	
C21	−0.0772 (4)	1.0202 (7)	0.1578 (4)	0.0329 (14)	
H21	−0.1328	1.0609	0.1686	0.039*	
C22	0.2969 (4)	1.1049 (9)	0.2789 (4)	0.0475 (18)	
H22A	0.2974	1.2154	0.2598	0.071*	
H22B	0.3511	1.0530	0.2649	0.071*	
H22C	0.2899	1.1040	0.3367	0.071*	

### Atomic displacement parameters (Å^2^)

**Table:**

	*U*^11^	*U*^22^	*U*^33^	*U*^12^	*U*^13^	*U*^23^
Ni1	0.0287 (5)	0.0273 (5)	0.0365 (5)	−0.0011 (3)	−0.0063 (3)	−0.0006 (3)
Br1	0.0521 (5)	0.0431 (5)	0.0457 (4)	−0.0035 (3)	0.0063 (3)	−0.0091 (3)
Br2	0.0549 (5)	0.0639 (6)	0.0688 (6)	0.0049 (4)	−0.0057 (4)	−0.0335 (4)
O1	0.034 (3)	0.038 (3)	0.045 (3)	−0.0006 (19)	−0.0068 (19)	−0.008 (2)
O2	0.034 (3)	0.063 (3)	0.059 (3)	0.001 (2)	−0.008 (2)	−0.021 (3)
O3	0.031 (2)	0.035 (2)	0.046 (3)	0.0019 (19)	−0.0116 (18)	−0.005 (2)
O4	0.036 (3)	0.038 (3)	0.060 (3)	0.001 (2)	−0.016 (2)	−0.012 (2)
N1	0.030 (3)	0.032 (3)	0.035 (3)	−0.006 (2)	−0.004 (2)	0.003 (2)
N2	0.030 (3)	0.029 (3)	0.039 (3)	−0.003 (2)	−0.006 (2)	0.005 (2)
C1	0.044 (4)	0.027 (3)	0.039 (4)	−0.003 (3)	−0.006 (3)	0.005 (3)
C2	0.030 (3)	0.028 (3)	0.043 (4)	−0.001 (3)	−0.009 (3)	0.005 (3)
C3	0.035 (4)	0.048 (4)	0.047 (4)	0.007 (3)	−0.007 (3)	−0.003 (3)
C4	0.030 (4)	0.074 (5)	0.056 (5)	0.005 (4)	−0.007 (3)	−0.004 (4)
C5	0.045 (4)	0.055 (5)	0.050 (4)	−0.004 (3)	−0.017 (3)	0.003 (3)
C6	0.036 (4)	0.046 (4)	0.039 (4)	−0.003 (3)	−0.011 (3)	−0.001 (3)
C7	0.036 (4)	0.028 (3)	0.035 (3)	−0.002 (3)	−0.006 (3)	0.005 (3)
C8	0.032 (3)	0.031 (3)	0.037 (3)	0.000 (3)	−0.005 (3)	0.002 (3)
C9	0.033 (4)	0.035 (4)	0.049 (4)	−0.007 (3)	−0.001 (3)	0.002 (3)
C10	0.032 (4)	0.036 (4)	0.044 (4)	0.003 (3)	0.006 (3)	0.001 (3)
C11	0.055 (5)	0.027 (3)	0.034 (4)	−0.001 (3)	0.002 (3)	0.000 (3)
C12	0.044 (4)	0.030 (3)	0.037 (4)	−0.007 (3)	−0.010 (3)	0.002 (3)
C13	0.044 (4)	0.025 (3)	0.038 (4)	−0.008 (3)	−0.012 (3)	0.002 (3)
C14	0.032 (4)	0.117 (8)	0.087 (6)	0.003 (5)	−0.011 (4)	−0.041 (6)
C15	0.036 (4)	0.025 (3)	0.034 (3)	−0.004 (3)	−0.003 (3)	0.001 (2)
C16	0.039 (4)	0.024 (3)	0.036 (3)	−0.003 (3)	−0.010 (3)	0.001 (3)
C17	0.030 (4)	0.029 (3)	0.050 (4)	0.002 (3)	−0.005 (3)	0.001 (3)
C18	0.043 (4)	0.040 (4)	0.045 (4)	−0.003 (3)	−0.012 (3)	−0.009 (3)
C19	0.047 (4)	0.039 (4)	0.038 (4)	0.002 (3)	−0.004 (3)	−0.009 (3)
C20	0.041 (4)	0.031 (4)	0.040 (4)	0.003 (3)	−0.001 (3)	−0.002 (3)
C21	0.029 (3)	0.030 (3)	0.040 (4)	−0.001 (3)	0.001 (3)	0.003 (3)
C22	0.036 (4)	0.045 (4)	0.061 (4)	−0.005 (3)	−0.018 (3)	−0.007 (3)

### Geometric parameters (Å, º)

**Table:**

Ni1—O3	1.852 (4)	C7—C8	1.418 (8)
Ni1—N2	1.859 (5)	C7—C13	1.418 (9)
Ni1—N1	1.861 (5)	C7—C12	1.423 (8)
Ni1—O1	1.863 (4)	C8—C9	1.429 (9)
Br1—C11	1.908 (6)	C9—C10	1.362 (9)
Br2—C19	1.915 (6)	C10—C11	1.402 (9)
O1—C8	1.288 (7)	C10—H10	0.9300
O2—C9	1.365 (7)	C11—C12	1.347 (9)
O2—C14	1.426 (9)	C12—H12	0.9300
O3—C16	1.302 (7)	C13—H13	0.9300
O4—C17	1.351 (7)	C14—H14A	0.9600
O4—C22	1.448 (7)	C14—H14B	0.9600
N1—C13	1.297 (8)	C14—H14C	0.9600
N1—C1	1.419 (8)	C15—C16	1.392 (9)
N2—C21	1.312 (7)	C15—C21	1.417 (8)
N2—C2	1.430 (7)	C15—C20	1.423 (8)
C1—C2	1.381 (9)	C16—C17	1.462 (8)
C1—C6	1.398 (8)	C17—C18	1.375 (9)
C2—C3	1.374 (9)	C18—C19	1.387 (10)
C3—C4	1.400 (9)	C18—H18	0.9300
C3—H3	0.9300	C19—C20	1.370 (9)
C4—C5	1.379 (10)	C20—H20	0.9300
C4—H4	0.9300	C21—H21	0.9300
C5—C6	1.369 (10)	C22—H22A	0.9600
C5—H5	0.9300	C22—H22B	0.9600
C6—H6	0.9300	C22—H22C	0.9600
			
O3—Ni1—N2	94.8 (2)	C9—C10—H10	120.2
O3—Ni1—N1	179.5 (2)	C11—C10—H10	120.2
N2—Ni1—N1	85.4 (2)	C12—C11—C10	121.7 (6)
O3—Ni1—O1	85.07 (18)	C12—C11—Br1	120.0 (5)
N2—Ni1—O1	179.8 (2)	C10—C11—Br1	118.3 (5)
N1—Ni1—O1	94.7 (2)	C11—C12—C7	119.2 (5)
C8—O1—Ni1	127.5 (4)	C11—C12—H12	120.4
C9—O2—C14	117.2 (5)	C7—C12—H12	120.4
C16—O3—Ni1	126.3 (4)	N1—C13—C7	126.6 (5)
C17—O4—C22	116.5 (5)	N1—C13—H13	116.7
C13—N1—C1	120.7 (5)	C7—C13—H13	116.7
C13—N1—Ni1	125.6 (4)	O2—C14—H14A	109.5
C1—N1—Ni1	113.7 (4)	O2—C14—H14B	109.5
C21—N2—C2	120.1 (5)	H14A—C14—H14B	109.5
C21—N2—Ni1	126.3 (4)	O2—C14—H14C	109.5
C2—N2—Ni1	113.6 (4)	H14A—C14—H14C	109.5
C2—C1—C6	119.3 (6)	H14B—C14—H14C	109.5
C2—C1—N1	113.9 (5)	C16—C15—C21	121.7 (5)
C6—C1—N1	126.8 (6)	C16—C15—C20	122.2 (5)
C3—C2—C1	121.2 (5)	C21—C15—C20	115.9 (6)
C3—C2—N2	125.4 (6)	O3—C16—C15	125.7 (5)
C1—C2—N2	113.4 (5)	O3—C16—C17	117.7 (5)
C2—C3—C4	119.2 (6)	C15—C16—C17	116.6 (5)
C2—C3—H3	120.4	O4—C17—C18	124.8 (5)
C4—C3—H3	120.4	O4—C17—C16	114.3 (5)
C5—C4—C3	119.5 (7)	C18—C17—C16	120.9 (6)
C5—C4—H4	120.3	C17—C18—C19	119.4 (6)
C3—C4—H4	120.3	C17—C18—H18	120.3
C6—C5—C4	121.3 (6)	C19—C18—H18	120.3
C6—C5—H5	119.4	C20—C19—C18	122.9 (6)
C4—C5—H5	119.4	C20—C19—Br2	118.2 (5)
C5—C6—C1	119.5 (6)	C18—C19—Br2	119.0 (5)
C5—C6—H6	120.2	C19—C20—C15	118.0 (6)
C1—C6—H6	120.2	C19—C20—H20	121.0
C8—C7—C13	120.8 (5)	C15—C20—H20	121.0
C8—C7—C12	121.3 (6)	N2—C21—C15	124.7 (6)
C13—C7—C12	117.9 (5)	N2—C21—H21	117.7
O1—C8—C7	124.7 (5)	C15—C21—H21	117.7
O1—C8—C9	119.6 (5)	O4—C22—H22A	109.5
C7—C8—C9	115.7 (6)	O4—C22—H22B	109.5
C10—C9—O2	123.7 (6)	H22A—C22—H22B	109.5
C10—C9—C8	122.4 (6)	O4—C22—H22C	109.5
O2—C9—C8	113.8 (6)	H22A—C22—H22C	109.5
C9—C10—C11	119.5 (6)	H22B—C22—H22C	109.5
